# Barriers to and Facilitators of Engagement With mHealth Technology for Remote Measurement and Management of Depression: Qualitative Analysis

**DOI:** 10.2196/11325

**Published:** 2019-01-30

**Authors:** Sara Simblett, Faith Matcham, Sara Siddi, Viola Bulgari, Chiara Barattieri di San Pietro, Jorge Hortas López, José Ferrão, Ashley Polhemus, Josep Maria Haro, Giovanni de Girolamo, Peter Gamble, Hans Eriksson, Matthew Hotopf, Til Wykes, RADAR-CNS Consortium

**Affiliations:** 1 Institute of Psychology, Psychiatry and Neuroscience King's College London London United Kingdom; 2 National Institute for Health Research Biomedical Research Centre for Mental Health South London and Maudsley National Health Service Foundation Trust King's College London London United Kingdom; 3 Parc Sanitari Sant Joan de Déu Sant Boi de Llobregat Centro de Investigacion Biomedica en Red CIBERSAM Madrid Spain; 4 Department of Psychiatry and Clinical Psychobiology University of Barcelona Barcelona Spain; 5 IRCCS Istituto Centro San Giovanni di Dio Fatebenefratelli Brescia Italy; 6 Department of Psychology University of Milano-Bicocca Milan Italy; 7 Research Department QITERIA Investigación Social Aplicada Madrid Spain; 8 Information Technology Department MSD Czech Republic Prague Czech Republic; 9 Clinical Development, Depression and Paediatrics H Lundbeck A/S Copenhagen Denmark

**Keywords:** acceptability, barriers, depression, facilitators, feasibility, mHealth, qualitative

## Abstract

**Background:**

Mobile technology has the potential to provide accurate, impactful data on the symptoms of depression, which could improve health management or assist in early detection of relapse. However, for this potential to be achieved, it is essential that patients engage with the technology. Although many barriers to and facilitators of the use of this technology are common across therapeutic areas and technology types, many may be specific to cultural and health contexts.

**Objective:**

This study aimed to determine the potential barriers to and facilitators of engagement with mobile health (mHealth) technology for remote measurement and management of depression across three Western European countries.

**Methods:**

Participants (N=25; 4:1 ratio of women to men; age range, 25-73 years) who experienced depression participated in five focus groups held in three countries (two in the United Kingdom, two in Spain, and one in Italy). The focus groups investigated the potential barriers to and facilitators of the use of mHealth technology. A systematic thematic analysis was used to extract themes and subthemes.

**Results:**

Facilitators and barriers were categorized as health-related factors, user-related factors, and technology-related factors. A total of 58 subthemes of specific barriers and facilitators or moderators emerged. A core group of themes including motivation, potential impact on mood and anxiety, aspects of inconvenience, and ease of use was noted across all countries.

**Conclusions:**

Similarities in the barriers to and facilitators of the use of mHealth technology have been observed across Spain, Italy, and the United Kingdom. These themes provide guidance on ways to promote the design of feasible and acceptable cross-cultural mHealth tools.

## Introduction

Depression is a major cause of disability in Europe and worldwide. It is associated with a range of negative outcomes including premature mortality [1], reduced quality of life [2], loss of occupational function [3], poor social integration and loneliness [4], and increased risk of other psychiatric problems such as comorbid anxiety disorders [5] and alcohol dependence [6]. Experiences of depression are commonly episodic, and the risk of recurrence following an initial episode is high [7].

With the global increase in availability of mobile phones and wearable devices [8,9], there is potential for more frequent health assessment that might help identify signals indicative of relapse, such as changes in behaviors, circadian rhythms, stresses, or symptoms [10]. Identification of such indicators might lead to fast treatment, possibly preventing relapse through early interventions [10]. However, a critical challenge is user acceptance of these technologies, particularly, the extent to which people are willing to engage with the technologies, considering the level of intrusiveness and possible discomfort. By engagement, we refer to the extent and manner in which people actively use resources. The level of engagement should specifically be important for people who are experiencing depression, as symptoms such as lack of motivation and interest to carry out activities (anhedonia) have been shown to influence the pursuit of potential rewards [11]. Clinical trials of mobile technologies for individuals with depression have highlighted engagement as a specific challenge [12].

In order to build on the potential of mobile technologies, we need to determine the views of people living with or having a history of depression, so that these views can be embedded at the start of the mobile health (mHealth) technology-designing process to ensure maximum applicability, acceptability, and adoption. This study builds on a recent systematic review of barriers to and facilitators of engagement with remote measurement technology [13]. This review used data from single-country studies, but engagement with mHealth technology may also be influenced by cultural context [14] in addition to individual differences. These differences would affect building of platforms that span across Europe and would need to be taken into account in the design of mHealth systems to maximize the value of interventions. This study aimed to identify these differences through focus groups from three European countries (Italy, United Kingdom, and Spain), providing an opportunity to identify a broader range of potential barriers to and facilitators of engagement and problems with adherence early in order to support the design of mHealth systems.

## Methods

### Design

A qualitative approach following a thematic analysis was employed to identify different experiences and potential barriers to and facilitators of engagement with mHealth technology among people with a history of or living with depression. The topic guide and coding frame were built on a recent systematic review on barriers to and facilitators of engagement with remote measurement technology [13]. Within the coding frame, several pre-established major and minor codes and subthemes emerged through the use of grounded-theory methods.

### Context

#### Researcher Characteristics

Native speakers in all countries managed the focus groups. Coordination among the three groups was agreed upon via telephone and email contact prior to commencing the study, and a facilitator with training in clinical psychology led each group. None of the facilitators were directly involved in the clinical care of the participants. All facilitators were female, apart from those in Spain, where the facilitators were a man and a woman. Notably, these characteristics may have influenced the collection and interpretation of data. To reduce some of this bias, the coding was replicated by a qualitative researcher who was not present in the focus group and did not have a background of clinical psychology. Disagreements in coding were resolved as a pair, and a joint decision was made about the allocation of a code to each quotation.

#### Participant Characteristics

Participants were eligible if they were above the age of 18 years and were currently experiencing clinically significant symptoms of major depressive disorder

or had experienced such symptoms in the past 2 years. Individuals with a history of a psychotic disorder, including bipolar disorder and schizoaffective disorder, and substance misuse in the last 6 months were excluded. Participants were recruited via different sources in the three countries. In the United Kingdom, potential participants were screened telephonically by using a self-report measure of depression (World Health Organization’s Composite International Diagnostic Interview - Short Form [15]). In Spain and Italy, clinicians selected patients diagnosed with major depressive disorder, who attended psychiatric services. Participants were identified by convenience sampling and their eligibility to participate. All participants provided written informed consent to participate in this study.

### Procedure

The local research ethics committees for each country approved the procedures (Ethics codes: United Kingdom, 16/LO/1513; Italy, Parere 5/2017; Spain, PIC-149-16). All participants were screened for their eligibility to participate over the phone or in person. Subsequently, they were invited to participate in a face-to-face focus group session. In this session, they first completed a consent form and a demographics questionnaire before participating in a focus group, as detailed below. All travel expenses were covered.

#### Focus Group

The discussion was semistructured using a prespecified topic guide (available on request) that was designed to elicit discussions about barriers to and facilitators of engagement with mHealth technology in the context of living with a long-term mental health condition. The open-discussion format allowed people to share a range of examples. Each group discussion lasted for 60-120 minutes. This format was developed and tested in the United Kingdom, where a second focus group with the same participants was conducted to validate the emerging findings.

#### Data Analysis

Focus group discussions were audio recorded and transcribed verbatim. Both the Italian and Spanish transcripts were translated into English, allowing combined analyses by two researchers working independently with the use of the software package NVivo (version 10; QSR International, Melbourne, Australia). Subthemes emerging from the data were identified in the final analysis.

## Results

### Participant Characteristics

Focus groups were conducted with 25 participants across three countries (United Kingdom, n=8; Spain, group 1: n=3, group 2: n=5; Italy, n=9). Participants in Spain and Italy were living with depression for longer than those in the United Kingdom, and all participants were Caucasian. In Spain, all participants were female, but the age of the participants was similar across all three countries (Table 1).

### Validation

Textbox 1 displays the subthemes emerging from the data, which were categorized into prespecified major and minor themes of the coding frame. Subthemes emerged in all major and minor codes of the coding frame, except physical ability. This evidence was taken as validation of the coding frame. Table 2 displays all the subthemes that emerged for the five different focus groups separately. Only a small number of additional subthemes emerged from the Spanish and Italian groups (10/58) after the focus group in the United Kingdom had taken place.

**Table 1:** Participant characteristics in each country.

Characteristics		United Kingdom (n=8)	Spain (n=8)	Italy (n=9)
Female, n (%)		5 (63)	8 (100)	7 (78)
Age (years), mean (SD)		51.9 (9.4)	47.1 (11.4)	52.8 (11.6)
Time since diagnosis (years), mean (SD)		2.9 (1.6)	13.2 (12.5)	11.5 (4.3)
**Ethnicity, n (%)**				
	White	5 (63)	8 (100)	9 (100)
	Black	2 (25)	—^a^	—
	Asian	1 (13)	—	—

^a^Not applicable.

**Final major and minor codes and subthemes emerging from the discussions**

**Health-related barriers and facilitators**

1. Symptom intensity or severity
  - Times of crisis
  - Accommodating fluctuations in symptoms
2. Emotional resources
  - Lack of motivation
  - Doubt
3. Awareness
  - Insight
4. Cognition
  - Poor memory (forgetfulness)
  - Difficulty reading
  - Difficulty with spoken expression
5. Physical ability

**User-related barriers and facilitators**

1. Technology acceptance
  - Attitude toward technology
  - Nonstigmatizing or familiar
  - Digital literacy (self and others)
  - Not ready to change
  - Codes of practice (eg, dress codes)
2. Perceived utility
  - Motivating action
  - Raising awareness or understanding
  - Sense of control
  - Opportunities for connection
  - Sense of achievement
  - Novelty or enjoyment
  - Measuring treatment response
  - Thinking more positively
  - Improving health and safety
  - Sharing data improves care
  - Reassuring (others)
  - Reassuring (others)
  - Contributing to research (others)
3. Perceived costs
  - Fears about privacy
  - Fears about security
  - Negative impact on mood or anxiety
  - Time and effort
  - Increased dependency
  - Fear of discrimination and stigma
  - Unavailable or burden on resources (others)
4. Overall value
  - Inaccurate, ineffective, or meaningless
  - Balancing utility and costs
  - Value of human contact
  - Managing expectations
  - Inability to sustain resources
  - Curiosity
  - Trust in experts

**Technology-related barriers and facilitators**

1. Convenience
  - Fitting with routine or lifestyle
  - Inconvenience of charging
  - Inconvenience of notifications
  - Automatic and simplifies life
  - Loss of connection
2. Accessibility
  - Tailored or personalized
  - Expense
  - Lacking equipment
3. Convenience
  - Ease of use
  - Wearable
  - Data visualization
  - Short assessments
  - Poorly designed systems
4. Intrusiveness
  - Passive data collection
  - Obtrusiveness or discomfort
  - Invasion of body

**Table 2:** Summary of themes across major and minor codes for all countries.

Theme		Group				
		UK (Group 1a)^a^, (n)	UK (Group 1b)^b^, (n)	Spain (Group 1), (n)	Spain (Group 2), (n)	Italy, (n)
**Health-related theme**						
	Symptom intensity or severity	Times of crisis (3)	—^c^	—	—	Times of crisis (1) Accommodating fluctuations (1)
	Emotional resources	Lack of motivation (2)	Lack of motivation (2)	Doubting benefits (1)	Lack of motivation (1)	Motivation as a moderator (3)
	Awareness	Insight as a moderator (2)	—	Poor insight (2)	Poor insight (3)	—
	Cognition	Forgetfulness (1)	—	—	Poor memory (2) Difficulty reading (3) Difficulty with spoken expression (1)	Forgetfulness (1)
**User-related theme**						
	Technology acceptance: self	Skepticism towards technology (4) Nonstigmatizing or familiar (1)	Skepticism towards technology (4) Nonstigmatizing or familiar (10) Digital literacy as a moderator (2) Not ready to change (1) Dress codes (1)	—	Familiar (1) Poor digital literacy (1)	Liking technology as a moderator (3)
	Technology acceptance: others	Lack of digital skills (2)	—	—	—	—
	Perceived utility: self	Motivating action (4) Raising awareness or understanding (4) Sense of control (4) Opportunities for connection (3) Sharing data improves care (2) Sense of achievement (1) Novelty or enjoyment (1)	Motivating action (6) Raising awareness or understanding (2) Sharing data improves care (1) Novelty or enjoyment (3) Measure treatment response (1) Thinking more positively (1)	Motivating action (1) Opportunities for connection (2) Sharing data improves care (1) Improves health and safety (1)	Sense of control (2) Sharing data improves care (2) Improves health and safety (2)	Reassuring (2)
	Perceived utility: others	Aiding decisions/communication (1)	Aiding decisions/communication (1) Contributing to research (8)	—	Aiding decisions/communication (1)	—
	Perceived costs: self	Fears about privacy (5) Negative impact on mood/anxiety (5) Fears about security and safety (3) Time and effort (1) Increased dependency (1)	Fears about privacy (4) Negative impact on mood/anxiety (3) Fears about security and safety (1) Time and effort (1) Fear of discrimination and stigma (7)	—	Negative impact on mood/anxiety (4)	Fears about privacy (5) Negative impact on mood/anxiety (1) Time and effort (2) Fear of discrimination and stigma (7)
	Perceived costs: others	Unavailable or burden on resources (4)	Unavailable or burden on resources (1)	—	—	Unavailable or burden on resources (1)
	Overall value	Inaccurate, ineffective, or meaningless (7) Balancing utility and costs (4) Value of human contact (2) Managing expectations (1) Inability to sustain resources (1)	Inaccurate, ineffective, or meaningless (6) Balancing utility and costs (3) Managing expectations (1) Sustainability of resources (2)	Inaccurate, ineffective, or meaningless (2) Balancing utility and costs (2) Curiosity (2)	Inaccurate, ineffective, or meaningless (1) Curiosity (2) Trust in experts (2)	Value of human contact (3)
**Technology-related theme**						
	Convenience	Fitting with routine/lifestyle (2)	Fitting with routine/lifestyle (9) Inconvenience of charging (1) Inconvenient notifications (1)	Inconvenience of charging (1) Inconvenient notifications (1) Automatic and simplifies life (2) Loss of connection (1)	—	Inconvenient notifications (3) Simplifies life (3) Loss of connection (3)
	Accessibility	Tailored or personalized (9)	Tailored or personalized (14) Expense as a moderator (2)	Expense as a moderator (3)	—	Tailored or personalized (2) Lacking equipment (1)
	Usability	Ease of use (3) Wearable (1) Data visualization (1) Short assessments (1) Poorly designed systems (1)	Data visualization (1)	Ease of use (1)	—	Ease of use (3)
	Intrusiveness	Passive data collection (4) Obtrusiveness (1) Live sharing (1)	Passive data collection (1) Obtrusiveness or discomfort (3) Invasion of body (1)	Obtrusiveness (1)	Discomfort (2)	—

^a^This group discussed prespecified points on the topic guide.

^b^This group reviewed topics raised in the first focus group to validate the findings.

^c^Not applicable.

### Barriers and Facilitators of Engagement

We present our results in 3 main categories: health-related barriers and facilitators, which included the impact of the health status of the individual on engagement with technology; user-related barriers and facilitators, which summarized the impact of user attitudes, preferences, and beliefs about engagement with technology; and technology-related barriers and facilitators, which focus on direct interaction with the technology.

#### 

##### 

Participants in the United Kingdom and Italy discussed the impact of depression on their ability to engage with mHealth technology. *Times of crisis* was the most difficult period to adhere to treatment; one participant mentioned, “at that stage you just don’t want to do anything. You’re just living in a self-imposed prison” (UK23). There may be a window of opportunity for clinical prediction:

Once you get over the edge, there is no going back. Until the wave passes, and then you get back to normality, but when we get to that stage, no advice, no nothing can help us, except ourselves.

UK23

Adjusting technology to *accommodate fluctuations in symptoms* may be important.

##### Emotional Resources

Lack of *motivation* was noted in all countries. Some participants spoke about reduced motivation during depression as “when I get in a downer, part of the issue is that I just cannot get on with anything” (UK24). In relation to remote measurement, one participant said, “I had to fill it in, in the morning, afternoon and evening. I did it for the first two days, then the third I did just at morning and afternoon and then stop, I didn’t do it anymore” (IT6).

##### Awareness

A subtheme of poor *insight* into the health status emerged across the United Kingdom and Spain; a participant stated, “I don’t always realise that I’ve suffered a dip or a rise” (SP8).

##### Cognition

The impact of difficulties with cognition that caused *problems with memory, reading, and expression* was only mentioned in Spain. Single participants in the United Kingdom and Italy mentioned that they might be forgetful, but they did not attribute this to cognitive difficulties.

#### 

##### 

###### Target Users

Participants’ general attitude towards mHealth technology emerged as a potential moderator of engagement. Participants in the United Kingdom demonstrated a *skeptical attitude:*

I don’t think for me personally technology would work, to be honest, because I’m a person more about feeling and touching, rather than kind of connecting with something cold things, and um, electronical

UK19

Acceptance may be influenced by *digital literacy*. One person was willing to accept technology with extra support, saying “you’d have to download the application for me because I don’t really know how those things work” (SP8). Others felt they had the required skills. Alternatively, some people *may not be ready to change* the way they manage their condition. This would be particularly important for individuals who do not own mobile technologies.

Acceptance of wearable devices that were *nonstigmatizing or familiar* was endorsed. One participant said, “I heard on TV that almost everyone nowadays has some sort of wearable device” (SP1); another participant added, “It wouldn’t be stigmatising. In fact, they’re quite trendy” (UK24). However, some participants raised concerns about employers not allowing people to wear devices due to *dress codes:*

I was just thinking about doctors and nurses and they’re not allowed to wear anything below their elbow.

UK22

###### 

UK participants discussed the impact of *digital literacy* amongst healthcare professionals, who may also find the use of new technologies difficult; one participant stated, “I worked with older GPs and they struggled with the new technology coming in” (UK18).

##### Perceived Utility

###### Target Users

Participants discussed aspects that would provide a utility and facilitate use. A function was deemed useful if the technology could *motivate action*, for example, “go for a walk...do some meditation” (UK2), or “call your doctor” (UK5):

I sometimes go out for a run and my phone tells me that it has detected physical activity. Of course, and when it picks up on that, it also tells me: you still have time to achieve your goal today

SP1

One participant in the United Kingdom said that this type of feedback might help to *think more positively*; another suggested that it could lead to a *sense of achievement*. Some thought that mHealth technology was *novel and enjoyable* besides useful.

*Raising awareness and understanding* of one’s health emerged as themes from the UK group. One person said “by measuring, you might discover things that people are not aware of already” (UK24). Feeling a *sense of control* and providing *opportunities for connection* with others may have further utility, as would using prompts or alerts to *improve health and safety* as, for example, a way to respond to symptoms early:

I have periods when I take medication and periods when I don’t well...until now, I’ve been the one to notice that oh, I’m not doing very well, or I’m a bit, I don’t know. And then after 3 or 4 weeks I’ve touched rock bottom. Well, maybe if I had some monitoring before that, then I could take the meds sooner and not get to that point, so, in my case, maybe it would be good for me

SP1

Sharing data with healthcare professionals was considered a way of *improving care* by this individual and others in the Spanish and UK groups. In the Italian group, health monitoring was considered *reassuring*.

###### 

Participants felt that there was scope for mHealth technology to *support clinical decision making and communication*; one participant said, “I could see that if um the tracking information would be useful for my doctor, to help with trying to find the right medication” (UK22). This view was shared across the United Kingdom and Spain. In addition, UK participants noted benefits of *contributing to research* and the potential wider impact on others with depression; one participant stated, “I know there’s a potentially bigger benefit—that’s worthwhile” (UK20).

##### Perceived Costs

###### Target Users

In addition to opportunities for utility, costs were identified. Participants feared about their *privacy and security:*

I don’t care if it knows I’ve been to Tesco’s this morning, don’t give a monkeys. But, I don’t particularly want people to know I’m in Tesco’s now.

UK20

I was given one of those new fashion ones, but I wanted one that looked cheap, otherwise I’m just going to get mugged

UK22

Although these issues were deemed important by some participants in Italy, one person mentioned, “I really don’t care about privacy.” [IT9]

Further costs were associated with feeling *increased anxiety* about one’s health:

The technology which could remind you, not remind you, but tell you that you’re going down or something. That would increase the anxiety, to be honest

UK23

I wouldn’t recommend it to a hypochondriac. Because they’d spend all day obsessed, keeping an eye on what’s happening to them.

SP5

I’m scared of relapses

SP6

Concerns about spending *time and effort* were mentioned by participants in Italy and the United Kingdom. In addition, there were concerns about *increasing dependency* and *fear of discrimination and stigma*. Participants suggested that data gathered might have “implications for travel insurance” (UK24) or prevent them from being promoted at work; one person stated, “I wouldn’t want to declare. I wouldn’t want to have a little badge on me saying I’m depressed.” (UK20).

###### 

The main area of concern was the *increased burden on resources* for healthcare professionals and its potential negative impact on care; one participant said, “The more that they’re bombarded with technology, the less energy there is for normal, human interaction” (UK18). Healthcare professionals and carers may not be *available to help* process information, and signs of deterioration may not be acted upon even if discovered.

###### Overall Value

People expressed *curiosity* about trying new technology. Hope for the future may provide motivation for engagement:

I’d quite happily do something that was two years, as long as I thought that if it was successful, there would be a hope for something afterwards

UK22

However, others in the UK group questioned the *sustainability of resources*, and the importance of *balancing utility and costs* was apparent. Investing money and time or making some sort of sacrifice to benefit from the rewards of the system was mentioned. One person felt that, overall, the perceived costs might outweigh the perceived utility. Due to current levels of information security, they said, “I think it is better not to collect this kind of personal data in the first place” (UK23). There were concerns across countries that the data gathered by the technology might be *inaccurate, ineffective, or meaningless*. Nonetheless, participants in Spain expressed *trust in researchers as experts* and were willing to be led by their guidance, but *managing participants’ expectations* of the achievements through remote measurement was highlighted as an important role of researchers in the United Kingdom.

UK and Italian participants emphasized on the *value of human contact*. In the Italian group, some participants raised general concerns about technology limiting the relationship with their clinician and preferred face-to-face contact rather than telehealth.

##### Convenience

Participants felt that technology played a role in *simplifying activities* and serving a purpose; one participant noted, “if there is a purpose, if it simplifies my life, I am glad to use it” (IT6). There were discussions about the pros and cons of wearing devices that doubled up as watches. The participants believed that technology should easily *fit within a daily routine*. Practical challenges were noted, such as losing opportunities to log data due to the appearance of *notifications at inconvenient timings*, the need for *charging*, and the *loss of connection*.

##### Accessibility

The *financial expense* associated with the devices was a potential moderator of accessibility, and practical issues including *lack of equipment* were considered a barrier. Even if technology was available, for it to be accessible, resources need to be *tailored or personalized* to meet the specific requirements of individuals. When a person feels more unwell than usual, this issue may affect usage. Comments such as “It would depend also on the severity of symptoms, it must be adjusted” (IT4) and “it must be tailored to the person’s mood and feelings” (IT8) highlighted this point.

##### Usability

mHealth resources should be *easy to use* and not “fiddly” (UK24). To reduce the effort needed to engage in surveys, one participant said, “I’d prefer something that is very short that I can complete within a minute” (UK20). Simplicity and low effort appear to be key facilitators, whereas complicated features or poor design were barriers:

I’ve got a watch my brother gave to me and it measures your heart rate. But it’s so sophisticated, you’ve got to stick a cable down here, it’s a bit much and I say: I’m thinking that I’m not going to wear this

SP3

*Wearable* monitors were endorsed, and the ability to *visualize data* was declared important for usability.

##### 

*Unobtrusive and comfortable* devices were important for acceptability. Similarly, discreet devices and *passive* collection of data were preferred. Only the UK group enquired about how *invasive devices* would be implanted under the skin.

A separate issue related to the theme of intrusiveness was the level of comfort participants felt with *live sharing* of data with others:

If its location, I’d rather it didn’t know, that data wasn’t live imported, instead when I’m not where the watch is telling, because you can get into live imports and just, everyone knows where you are all the time. And some of my cousins are quite happy to know where each other are 24/7, I find that scary… horrible, I don’t want that.

UK20

Knowing who the data would be shared with was deemed important, and some participants suggested that sharing data with clinicians may be more acceptable than sharing them with profit-driven organizations.

### Cross-Country Comparisons

Almost half the themes were similar across at least two countries, suggesting replication and an acceptable level of data saturation [16]. A core group of themes was repeated across all countries: the need for *motivation* **,** the potential *negative impact on anxiety and mood*, the inconvenience of *too-frequent notifications*, and the importance of *ease of use.* A number of key differences regarding additional subthemes emerged between regions. First, although the UK group provided an extensive list of utility examples, they were skeptical about the use of mHealth technology. Similarly, the Spanish participants had many issues with perceived utility. In contrast, the Italian group focused more on perceived costs. Participants in Spain were the only group to trust the experts. The UK group was uncertain about the digital skills and availability of resources in clinical practice. Acceptance of technology from the perspective of other people in their health systems, such as clinicians, was not raised as a concern in Spain or Italy. One older participant textboxin Italy expressed the inability to access equipment. Second, issues regarding usability were discussed in greater depth in the UK group. Although the ease of use was the only subtheme in the Italian and Spanish groups, some specific suggestions about data visualization, length of assessments, and the ease of wearables emerged in the UK group. Few technology-related barriers and facilitators emerged in Spain, where participants focused more on health-related and user-related themes than technology-related themes.

## Discussion

### Principal Findings

**Figure 1:**
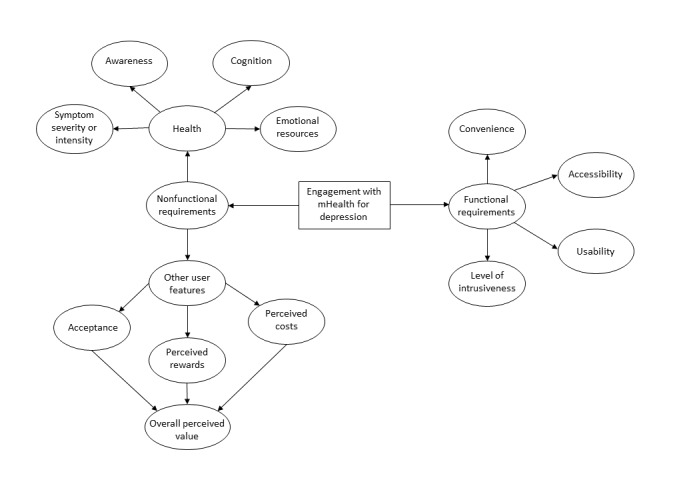
Requirements for engagement with mobile health (mHealth) technology for depression.

In this study, 3 major, 14 minor, and 58 subthemes emerged from the data; some were related to functionality of technology and others were about users’ abilities, perceptions, and attitudes toward technology. These nonfunctional requirements have been reported previously [17]. Our nonfunctional requirements were categorized as health-related and user-related barriers and facilitators (Figure 1).

### Nonfunctional Requirements

In terms of health-related barriers and facilitators, the severity of symptoms may moderate engagement with mHealth technology. Low motivation and intermittent poor insight and memory are known to be symptoms of depression and may be specific to this population [18], which may affect mHealth systems that require direct interaction with an app as well as users’ decisions to wear devices. However, the absence of data could be as informative as its presence in algorithms created to identify the risk of relapse.

In terms of user-related barriers and facilitators, participants’ attitudes toward mHealth technology affected their engagement. Similarly, digital literacy moderates the use of technology [19], and as the results of this study suggest, affects all users including the healthcare providers who support patients. Familiarity with technology and employment regulations may either further facilitate technology adoption or pose a barrier for use.

Our participants emphasized the importance of weighing costs against utility in order to make a decision about the overall value of mHealth technology. Utility included factors such as opportunities to connect with others; prompts; raising awareness and understanding; a sense of control; and responding to early warning signs by, for example, supporting clinical decision making. Perceived costs included reduced privacy and security; lack of availability and limited resources to support use; increased health anxiety or dependency; and expending time and effort, especially if there were inaccuracies in measurement.

### Functional Requirements

Previous research demonstrated a relationship between perceived convenience and usability, and the acceptance of technology [20]. Similarly, mHealth technologies were thought to be easier to accept if they reduced effort, served a clear purpose, fit into one’s daily routine, were comfortable, and promoted choice or control. Barriers included the receipt of notifications at inconvenient times and the need to charge devices or fix technical malfunctions. In addition to convenience and usability, previous literature has advocated the development of resources that are accessible or equally available to all users (eg, “universal design”) [21], and this work has reiterated the need for such development with respect to depression-specific symptoms.

### Geographical Requirements

The subthemes that emerged from multiple countries demonstrate some of the most important considerations for developing mHealth resources across Europe. *Motivation* is a key moderator of engagement. Two barriers across countries were the *potential negative effect on anxiety and mood* and the *inconvenience of too-frequent notifications* *,* which may be related. Focus on an *easy-to-use design* was clear. Some differences between the countries may relate to diversity in health care experience and the availability of or familiarity with mHealth technology. There is variation in the percentage of adults using mobile phones and internet-based technologies across Europe; Italy has lower access to these technologies than Spain and the United Kingdom [22]. Varied familiarity with mHealth technology may account for fewer examples of utility and greater concerns about potential costs in Italy, where some people may not be fully aware of the benefits and may have raised potential concerns about the loss of human interaction.

Results from this study are similar to those of a systematic review on barriers to and facilitators of engagement with remote measurement technology [13]. However, our study focused on the attitudes of individuals with depression toward technology and the nonfunctional, rather than functional, factors. Motivation was clearly an important category, but was incorporated into health-related barriers and facilitators in this study, due to the inextricable link between mood and emotional resources for people with depression; their physical abilities were never discussed. Although a few previous studies reported the acceptability and feasibility of mHealth resources for people with mental health conditions (eg, [23-26]), none of them explored barriers and facilitators across several countries.

This study uniquely provides views from participants living in different countries and revealed both similar and potentially different issues that were considered by the different groups. Although mHealth resources should take into account the similarities of views, it is essential to continue monitoring engagement across different countries, as these differences may affect their efficient implementation.

### Strengths and Limitations

Strengths of this study were the inclusion of a varied sample that represented three European countries that place similar emphasis on community-based or “remote” treatment interventions for mental health. The qualitative approach enabled a rich, in-depth discussion of possible barriers to and facilitators of engagement with mHealth technology. It was not constrained to responses to specific questions, which allowed the discovery of themes that may not have emerged otherwise.

Although another strength of the study was the cross-national approach to understand factors influencing engagement, it is important to note that the translation may have influenced the findings. A further key limitation is the dependence of our results on hypothetical scenarios rather than actual experience. We have identified several themes that can guide research design and technological development, but we should be cautious about the anticipated risks or benefits that may not be sustained when people are exposed to technology. Further user testing with specific prototypes is required to maximize acceptability and usability. Such user testing will include a wider sample of the population with a history or current symptoms of depression, which will involve purposive sampling.

### Future Research

Future research should consider other stakeholders. Craven et al [17] advocate the involvement of all possible end users including carers and clinicians, which may result in systems that are easily implemented in practice. A few studies that implemented this holistic perspective and involved several users [27] found commonalities in terms of universal support for technology innovation and potential barriers to the use of mHealth technology, similar to those identified in this study.

### Conclusion

This qualitative study investigated the potential barriers to and facilitators of engagement with mHealth technology. A number of functional and nonfunctional categories emerged with both similarities and differences across European countries. The themes form a platform for future research on engagement with mHealth technology as a part of healthcare. A number of hypotheses have been generated: Increased familiarity and perceived utility, improved choice and control, greater convenience and accessibility, and lower intrusiveness may influence decisions about the use and engagement of mHealth technology and should be encouraged and evaluated in future studies, as the data might provide useful to improve existing models.

## Abbreviations

mHealth: mobile health
